# Evaluation of calpain T-cell epitopes as vaccine candidates against experimental *Leishmania major* infection: a pilot study

**DOI:** 10.1007/s00436-022-07657-7

**Published:** 2022-09-14

**Authors:** Reham Brakat, Amal Mahmoud, Eman Abd El Gayed, Shaimaa Soliman, Shaimaa Sharaf-El-Deen

**Affiliations:** 1 Faculty of Medicine, Menoufia University, Shibin El Kom, Egypt; 2 Department of Biology, College of Science, Imam Abdulrahman Bin Faisal University, P.O. Box 1982, Dammam, 31441 Saudi Arabia

**Keywords:** Leishmania major, Vaccine, Calpain, T-Cell epitope, Memory cells

## Introduction

Leishmaniasis is a neglected tropical disease caused by *Leishmania* (*L.*) species. Infection usually occurs due to the bite of an infected sand fly. Leishmaniasis manifests in a variety of pathological forms depending on the infecting species, ranging from fatal systemic disease (visceral leishmaniasis) to localized skin ulcers (cutaneous leishmaniasis [CL]), which is the most common form (Kaye et al. 2020). According to the World Health Organization, 92 countries are considered endemic for CL or have previously reported cases, with more than one billion people inhabitants at risk of infection. The number of new cases reported annually is more than one million (https://www.who.int/leishmaniasis/en/).

The CL ulcers commonly form on exposed skin areas of the body and usually heal spontaneously, leaving permanent disfiguring scars. This disfigurement is a cause of psychological distress, social stigma, and decreased quality of life. When CL occurs in individuals from nonendemic areas, such as travelers and military personnel, the problem becomes more complicated because it is frequently misdiagnosed as a form of skin ulcer. Thus, the patient loses the opportunity to get proper treatment at the appropriate time (Ennes-Vidal et al. 2017; Bilgic-Temel et al. 2019).

Control of CL depends primarily on chemotherapeutic agents that have many side effects and may not be effective against the emergence of resistant strains. For this reason, vaccination is a logical step toward the proper management of CL (Rabienia et al. 2020). Due to the lifelong immunity that results from natural infection, developing an effective vaccine for the global eradication of CL is an attainable goal (Gillespie et al. 2016; Zhang et al. 2020).

Epitope-based vaccines have shown promising results among various vaccine approaches. They are based on short antigenic epitopes that can elicit the desired immune response and stimulate specific CD4 + and CD8 + T-cell responses, which are crucial for clearing *Leishmania major* infection (Skwarczynski and Toth 2016; Bordbar et al. 2020). In terms of ease of manufacture, low cost, absence of potentially harmful substances, reduced antigen complexity, and stability, epitope-based vaccines outperform other vaccination strategies. One of the primary considerations in selecting a target gene is the ability of its protein product to elicit a robust immune response (Gershoni et al. 2007; Skwarczynski and Toth 2016; De Brito et al. 2018).

Calpains are specific calcium-dependent cysteine proteases expressed by several parasites, such as *Trypanosomes*, *Leishmania*, *Schistosomes*, and *Plasmodium falciparum* (Kumar and Ahmad 2017). In *L. major*, 27 calpain sequences have been identified, which are thought to be involved in essential processes (Ersfeld et al. 2005; Branquinha et al. 2013). Calpain upregulation has also been associated with drug resistance and virulence in *Leishmania* (Ennes-Vidal et al. 2017). Therefore, they were tested as targets for vaccine and drug development. The therapeutic efficacy of calpain inhibitors was found to be satisfactory and reduced promastigote proliferation and the number of intracellular amastigotes (De Souza Araújo et al. 2018). To our knowledge, this is the first published study evaluating calpain peptides as vaccine candidates for protection against *L. major.*

In this study, we used immunobioinformatics tools to design six T-cell epitopes from *L. major* calpains directed at CD4 + or CD8 + T-cells. In addition, we aimed to evaluate the protective effects of these epitopes adsorbed to the Imjet alum adjuvant and the mechanism of this protection in BALB/c mice infected with *L. major* promastigotes.

## Methods

### Ethics statement

Mice were kept under standard breeding conditions in the animal house of Theodor Bilharz Research Institute (TBRI), Giza, Egypt. The breeding room was air-conditioned at 20–22 °C, and mice were fed a commercial pellet diet. All the study procedures were performed in accordance with the international ethical guidelines approved by the institutional ethical committee of TBRI and Faculty of Medicine, Menofia University (ethics number, 9/2021PARA20).

### Epitope prediction

The FASTA-formatted amino acid sequences of calpain-like cysteine peptidases were retrieved from the National Center for Biotechnology Information (NCBI) database. The VaxiJen v2.0 server (http://www.ddg-pharmfac.net/vaxijen/VaxiJen/VaxiJen.html) was used to predict the sequence of the most antigenic calpain-like cysteine peptidase with a threshold of 0.5 (Doytchinova and Flower 2007). T-cell epitopes were predicted using IEDB (http://tools.iedb.org/mhci/ and http://tools.iedb.org/mhcii/), with an IC50 binding value < 200 and < 1000 for MHC-I and MHC-II, respectively). The conservancy (http://tools.iedb.org/conservancy/), with sequence identity threshold 100%, and immunogenicity (http://tools.iedb.org/immunogenicity/) prediction tools of IEDB were used for epitope conservancy and immunogenicity predictions, respectively (Li et al. 2008; Calis et al. 2013). The population coverage tool (http://tools.iedb.org/population/) was used to analyze the population coverage of the predicted epitopes and their respective MHC HLA-binding alleles (Dimitrov et al. 2014a). Allergenicity was predicted using AllerTOP v. 2.0 (https://www.ddg-pharmfac.net/AllerTOP/index.html) (Dimitrov et al. 2014a) and AllergenFP 1.0 (http://www.ddg-pharmfac.net/AllergenFP/) (Dimitrov et al. 2014b). Peptide toxicity was predicted using ToxinPred (http://www.imtech.res.in/raghava/toxinpred/) (Gupta et al. 2013). BepiPred-2.0, with a threshold of 0.5 (https://services.healthtech.dtu.dk/service.php?BepiPred-2.0) (Jespersen et al. 2017), and LBtope, with % probability threshold of 60% (http://crdd.osdd.net/raghava//lbtope/) (Singh et al. 2013), were used to identify B-cells with the most antigenic proteins. The designated short peptides were synthesized by GenScript, USA.

### Animals and study design

A total of 115 inbred pathogen-free male BALB/c mice (6–8 weeks, 18–20 gm) were randomly categorized into three groups. Group I (GI; 10 mice) served as the adjuvant (uninfected) control. Group II (GII; 15 mice) served as the *L. major*-infected control. Group III (GIII; 90 mice) was the T-cell epitope-immunized group. Based on epitope type, GIII was further classified into subgroups A–F. Each subgroup included 15 mice.

### Immunization protocol

GIII mice were subcutaneously injected with 100 µg of the synthetic peptide in the right footpad. The immunization dose was prepared by dissolving 100 µg of the synthetic peptide in 100 µL of phosphate buffered saline (PBS) (Spitzer et al. 1999), then adding 100 µL of Imject Alum (an aqueous solution of aluminum hydroxide and magnesium hydroxide plus inactive stabilizers; Thermo Fisher Scientific, USA), and vortexing the solution for 30 min to allow the Imject Alum to effectively adsorb the antigen. A similar booster dose was administered 3 weeks later. The synthetic peptides received by subgroups A–F are summarized in Table 1. GI mice were subcutaneously injected with two doses of 100 µL of Imject Alum alone with 3-week interval.

**Table 1:** Summary of epitope sequences used for the immunized GIII

Subgroup name	Targeted lymphocyte	Synthesized peptide
A	CD4 +	NERARELAWATLAAD
B	CD4 +	DPAKHAGAIAHLESE
C	CD4 +	TQHPVLLRRLIVTKE
D	CD8 +	ARELAWATL
E	CD8 +	KHAGAIAHL
F	CD8 +	LLRRLIVTK

### Challenge with *L. major*

Three weeks after the booster dose, GII and GIII mice were subcutaneously injected with 2 × 10^6^ *L. major* promastigotes (in 50 μL PBS) (MHOM/IL/81/FEBNI) into the left footpad. This strain was obtained from the Faculty of Veterinary Medicine, Cairo University, Egypt, where the infection was maintained in laboratory bred BALB/c mice. Promastigotes were harvested from the Novy–MacNeal–Nicolle medium during the stationary phase.

### Evaluation of skin lesions

Two weeks after *L. major* challenge, the sizes of the developing footpad swellings were measured weekly using digital calipers until week 8 post infection (p.i.). The thickness and width of the left and right footpads were measured in millimeters, and lesion size was calculated according to the following equation (Shermeh et al. 2021).

$$\frac{\left(\mathrm{left\;foodpad\;thickness }+\mathrm{ left\;footpad\;width}\right) - (\mathrm{right\;foodpad\;thickness }+\mathrm{ right\;footpad\;width})}{2}$$

### Outcome measures

Mice groups were assessed by TCM flowcytometry (3 weeks after the booster immunization; Zhang et al. 2013), parasite cycle threshold (CT) (Tupperwar et al. 2008), Th1 [IgG2a and IFN-γ] and Th2 biomarkers [IgG1 and IL-4] (4 weeks p.i.), and lesion size measurement (from week 2 to 8 p.i.; Shermeh et al. 2021).

### Flow cytometric analysis of TCM lymphocytes

Three weeks after the booster dose, five mice from each group (except 3 from GI) were euthanized by cervical dislocation. Popliteal lymph nodes were dissected and pooled in RPMI 1640 medium (Sigma-Aldrich, USA) and pressed through a 70-µm cell strainer on top of a 15-mL tube with a sterile 3-mL syringe plunger. The lymph nodes were frequently flushed with 6 mL RPMI, containing 10% fetal bovine serum. The collected cells were centrifuged at 500 × *g*, 5 min. After the supernatant was discarded, the pellet was resuspended in 500 µL PBS and refiltered using a 30-µm cell strainer into a new 15-mL tube to remove coagulated cells. Then, we performed flowcytometric analysis of T central memory (TCM) cells (Haubruck et al. 2020). TCM cells of popliteal lymph nodes were labeled with anti-CD4, CD44, and CD62L monoclonal antibodies (Abcam, USA). Cell percentage was determined using the fluorescence-activated cell sorter Calibur system (Becton Dickinson Immunocytometry Systems, San Jose, CA, USA) (Zhang et al. 2013).

### Quantification of parasitic burden using real-time polymerase chain reaction (RT-PCR)

Four weeks p.i., 5 mice from each group (except 3 from GI) were decapitated. Popliteal lymph nodes were dissected and frozen to measure the parasitic burden using RT-PCR. Genomic DNA was extracted from 10 mg frozen lymph nodes of each mouse using the JET Genomic DNA Purification Mini Kit (Thermo Scientific, EU/Lithuania). The extracted DNA was stored at − 20 °C until use. Primer specificity was confirmed using Primer BLAST. Each primer (25 nmole) was dissolved in 250 μL RNase-free water to obtain a solution of 100 μmol/L as a final concentration. The following primers were used: forward, 5′-GGG GTT GGT GTA AAA TAG GG-3′ and reverse 5′-TTT GAA CGG GAT TTC TG-3′ (Miland, TX). A total volume of 20 µL was used for the RT-PCR reaction, including 10 μL SYBR Green 2 × QuantiTect PCR Master Mix (Thermo Fisher Scientific, Waltham, USA), 3 μL DNA, 1 μL forward primer, 1 μL reverse primer, and 5 μL RNase-free H_2_O. The reaction mix was incubated at 95 °C for 10 min, followed by 40 cycles of denaturation at 95 °C for 10 s, annealing at 60 °C for 15 s, and extension at 72 °C for 20 s. Data analysis was performed using the Applied Biosystems 7500 software version 2.0.1 in the Central Lab, Faculty of Medicine, Menoufia University. Fluorescence was measured in the green channel, and data were collected during the extension step. CT values were individually calculated with an internal software using a manual threshold setting of 0.2 and activating the “dynamic tube” and “ignore first 10 cycles” functions. After each run, melt-curve analysis was performed to match amplicons with positive control melt-curve peaks and confirm specificity. CT value in the lymph node was inversely proportional to the amount of DNA detected by RT-PCR.

### Assessment of IFN-γ and IL-4

A part of the popliteal lymph node obtained 4 weeks p.i. was homogenized according to Belkhelfa-Slimani and Djerdjouri (2018). Briefly, lymph nodes were homogenized in 50 mM ice-cold phosphate buffer (pH = 7.2), containing 0.5% Triton X-100 (Sigma-Aldrich, USA), and freeze-thawed three times. Homogenates were centrifuged at 10,000 × *g* for 10 min at 4 °C. Supernatants were stored at − 70 °C. Levels of IFN-γ and IL-4 in the supernatant of the lymph node homogenate were measured using solid-phase sandwich ELISA (Life Technologies Corporation, Thermo Fisher, USA for IFN-γ and Abcam, USA for IL-4). The procedures were performed according to the manufacturer’s protocol.

### Assessment of antibody response (IgG2a and IgG1)

Four weeks p.i., serum of decapitated mice was used to measure IgG2a and IgG1 levels. Stationary promastigotes suspended in PBS underwent three cycles of freezing and thawing and then centrifuged at 12,000 × *g* for 15 min. The supernatant containing the soluble *Leishmania* antigen (SLA) was collected, and the protein concentration was measured by colorimetry, adjusted to 1.3 gm/dL, and stored at − 70 °C until use (Solana et al. 2017). A titration curve was constructed, and the optimum concentration of SLA was found to be 200 ng/well. Then, 96-well microtiter plates were coated with SLA and incubated at 37 °C for 4 h. The plates were washed and blocked overnight at 4 °C with 200 μL of 10% fetal calf serum in PBS-Tween per well. Serum samples were diluted to 1:100 with PBS-Tween-10% fetal calf serum and applied to plates in twofold serial dilutions. Plates were incubated at 37 °C for 4 h, washed, and incubated with 1:2000 dilution of horseradish peroxidase-conjugated goat anti-mouse IgG2a or IgG1 antibodies (Zymed Laboratories Inc., USA) for 2 h at 37 °C. Detection was performed using the colorimetric substrate 3,3′,5,5′-tetramethylbenzidine (Sigma-Aldrich). Optical density (OD) was read at 450 nm.

### Statistical analysis

Statistical analysis was performed using the SPSS statistical package, version 26 (SPSS Inc. Released 2019. IBM SPSS statistics for windows, version 23.0, Armnok, NY: IBM Corp.). Variables were expressed as the mean (x̅), standard deviation, median, and range. Analysis of variance (ANOVA) (with homogeneity testing) was used for comparison of quantitative variables between more than two groups of normally distributed data with Tukey’s post hoc test. The Kruskal–Wallis test was used for comparison of quantitative variables between more than two groups of abnormally distributed data with the Tamhane post hoc test. A two-sided *p* value of < 0.05 was considered statistically significant.

## Results

### Calpain-like cysteine peptidase epitope prediction

**Fig. 1:**
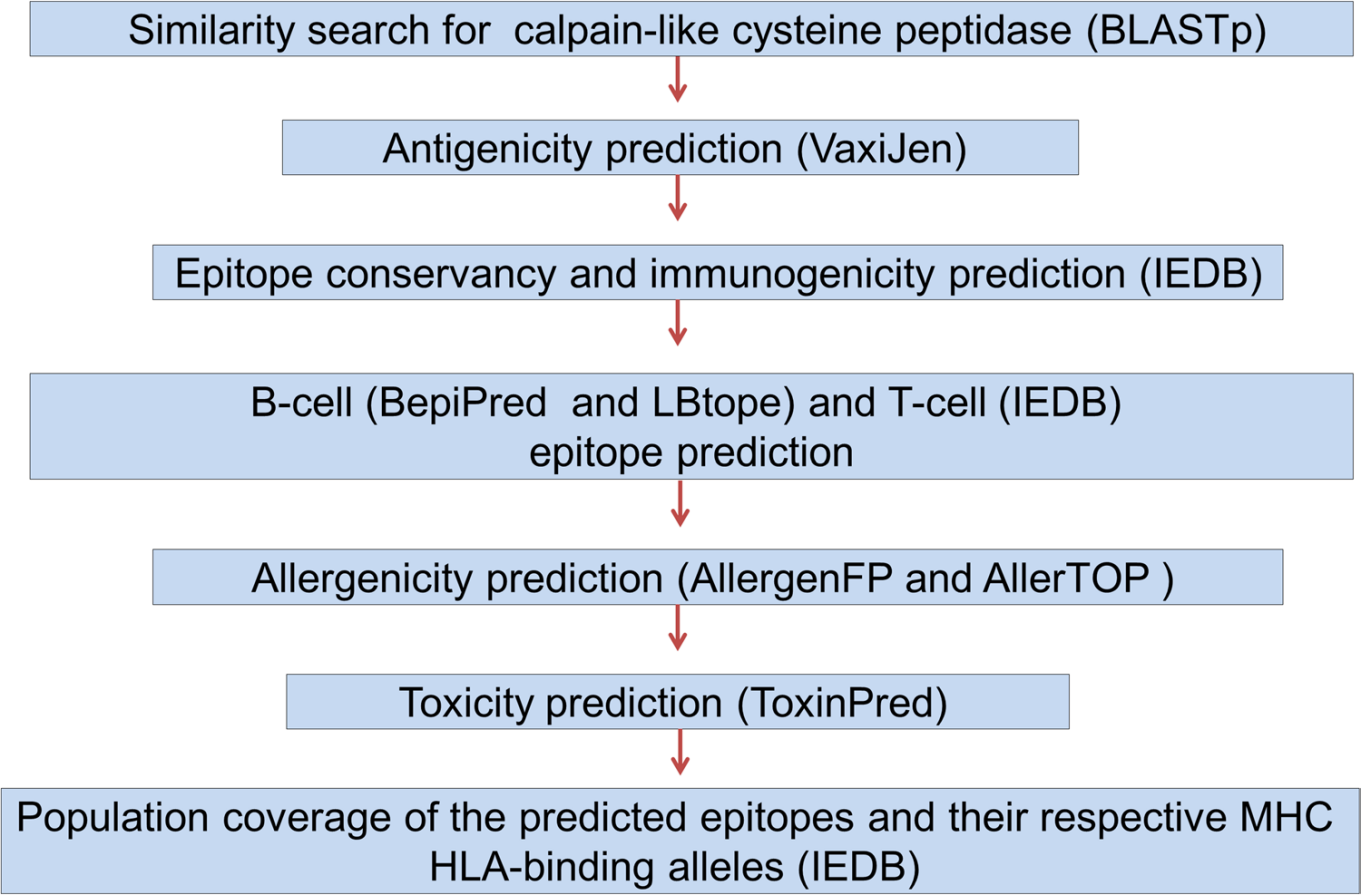
Flow chart depicting in silico vaccine design strategies against *Leishmania major*

The query for calpain-like cysteine peptidase XP_003721857.1 (5,358 aa) was retrieved from UniProt and used for database search using BLASTp. A total of 100 hits (identity 34.12–91.52%) in the NCBI database, eight highly similar sequences (identity 76.25–91.52%), and XP_003721857.1 were used. Among all calpain-like cysteine peptidase sequences, the protein sequence with GenBank ID > TPP47601.1 had the highest antigenic score of 0.5901. This protein was used for further analysis (Fig. 1).

Of the 203 predicted CD8 + T-cell epitopes, we identified three (ARELAWATL, KHAGAIAHL, and LLRRLIVTK) that were characterized by high antigenicity and immunogenicity scores, conservancy, and allergenicity (Table 2). These epitopes were used to predict MHC-II alleles and their respective peptides or CD4 + T-cell epitopes (Table 2).

**Table 2:** Predicted MHC-I and MHC-II epitopes

MHC-I epitopes	Antigenicity (threshold 0.5)	Immunogenicity	Allergenicity	Toxicity (threshold 0.75)	MHC-I interaction (affinity of IC50 < 200)	MHC-II epitopes	MHC-II interaction (affinity of IC50 < 1,000)
ARELAWATL	0.2611	0.32365	Non-allergen	Non-Toxin	HLA-A*02:50, HLA-A*32:07, HLA-C*03:03, HLA-C*12:03, HLA-B*58:01, HLA-B*15:02, HLA-C*07:02, HLA-A*32:15, HLA-B*57:01, HLA-C*07:01	NERARELAWATLAAD	HLA-DRB1*04:05
KHAGAIAHL	0.7	0.2621	Non-allergen	Non-Toxin	HLA-A*02:50, HLA-C*03:03, HLA-A*32:07, HLA-B*15:02, HLA-A*32:15, HLA-C*12:03, HLA-A*02:17, HLA-B*57:01, HLA-A*02:17	DPAKHAGAIAHLESE	HLA-DQA1*01:02 HLA-DQB1*06:02
LLRRLIVTK	0.496	0.24088	Non-allergen	Non-Toxin	HLA-A*32:07, HLA-C*12:03, HLA-A*31:01, HLA-A*01:01	TQHPVLLRRLIVTKE	HLA-DRB1*04:05 HLA-DRB1*08:02 HLA-DRB1*04:01

### Effects of epitopes on TCM cells

**Fig. 2:**
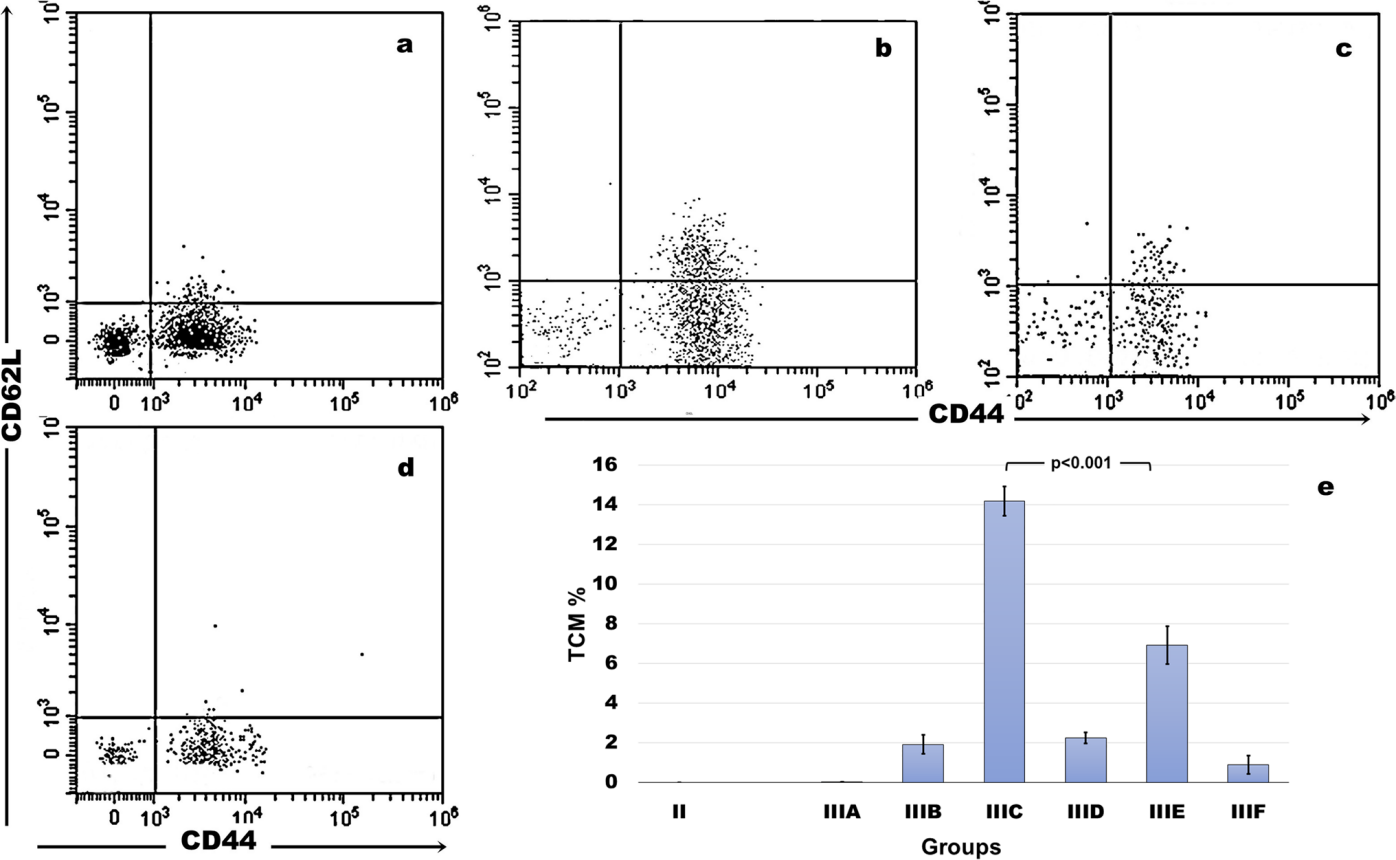
**a** Flow cytometry plot of CD44 + CD62L + T central memory (TCM) cells of subgroup B showing decreased TCM percentage. **b** Flow cytometry plot of CD44 + CD62L + TCM cells of subgroup C showing the highest TCM percentage compared with other subgroups. **c** Flow cytometry plot of CD44 + CD62L + TCM cells of subgroup E, which ranked second to subgroup C in TCM percentage. **d** Flow cytometry plot of CD44 + CD62L + TCM cells of subgroup F showing markedly decreased TCM percentage.** e** Column chart presentation of the mean TCM percentages of the studied groups. The highest TCM percentages were detected in subgroups C and E, with statistically significant differences compared with other groups

The highest percentage of TCM cells was detected with the CD4 + lymphocyte-directed TQHPVLLRRLIVTKE epitope used in subgroup C (14.20% ± 0.73%). The CD8 + lymphocyte-directed epitope, KHAGAIAHL used for subgroup E, also elicited a marked elevation of TCM cells (6.92 ± 0.95) and ranked second to subgroup C, with a statistically significant difference between both subgroups (*p* < 0.001). TCM cell percentages of the remaining subgroups were markedly lower than subgroups C and E, with statistically significant differences (*p* < 0.01). The lowest percentage was recorded in subgroup A (0.02% ± 0.01%) (Fig. 2).

### Disease progression and lesion size

**Fig. 3:**
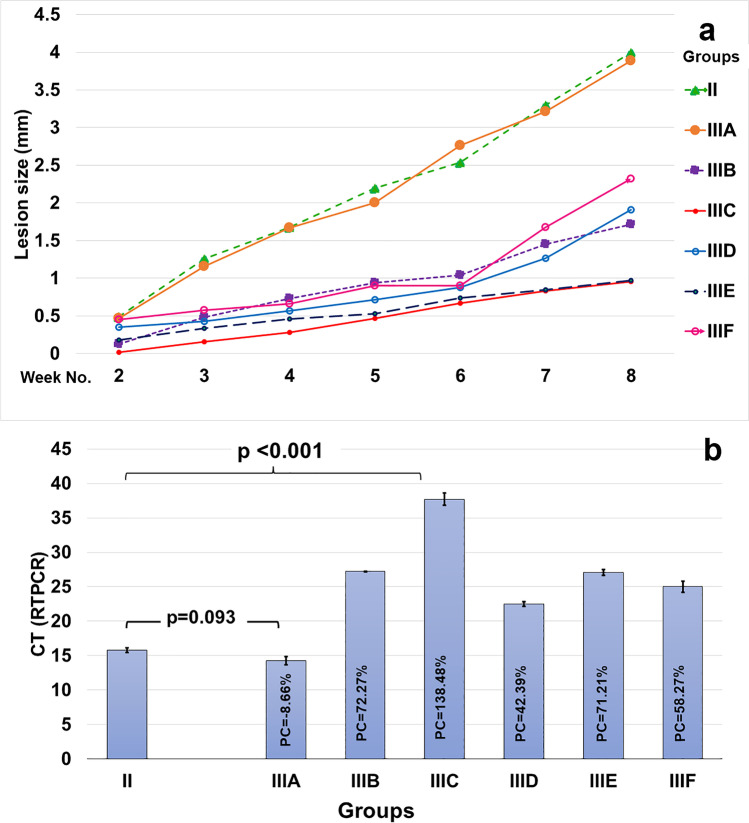
**a** Curve presentation of progression of lesion size in the studied groups during the study. The smallest sizes were detected in subgroups C and E. **b** Column chart presentation of mean CT values of the studied groups. The highest CT (i.e., lowest parasite burden) was detected in subgroups C and E, with statistically significant differences compared with the infected control (GII) and other subgroups. The lowest CT (i.e., highest parasite burden) was detected in subgroup A, which was not significantly different from the nonvaccinated infected control

All the vaccinated mice showed milder disease progression compared with the infected controls, except those in subgroup A. The slowest disease progression and smallest lesion size throughout the study were detected in subgroup C mice, followed by subgroup E mice, with statistically significant differences compared with the other subgroups (*p* < 0.05). The progression of the disease in subgroup A was not much better than that in the infected control. The difference between both groups was not statistically significant (*p* > 0.05) (Fig. 3a).

### Effects of epitopes on parasite burden

Subgroup C, immunized with the CD4 + lymphocyte-directed epitope TQHPVLLRRLIVTKE, had the highest CT value (37.70 ± 0.89), suggesting lowest parasite burden with a percentage of change (PC) = 138.48%. Differences between subgroup C and all other subgroups were statistically significant (*p* < 0.001). Subgroups E (27.07 ± 0.39; PC = 71.21%) and B (27.24 ± 0.04; PC = 72.27%), which received CD8 + and CD4 + lymphocyte-directed epitopes, KHAGAIAHL and DPAKHAGAIAHLESE, respectively, were ranked second (*p* = 1.00). Subgroup A was ranked last (14.24 ± 0.59; PC =  − 8.66). In contrast to that of the other immunized subgroups, the CT of subgroup A was not statistically different from that of the infected control group II (15.82 ± 0.34) (*p* = 0.093) (Fig. 3b).

### Effects of epitopes on levels of IFN-γ and IL-4

**Fig. 4:**
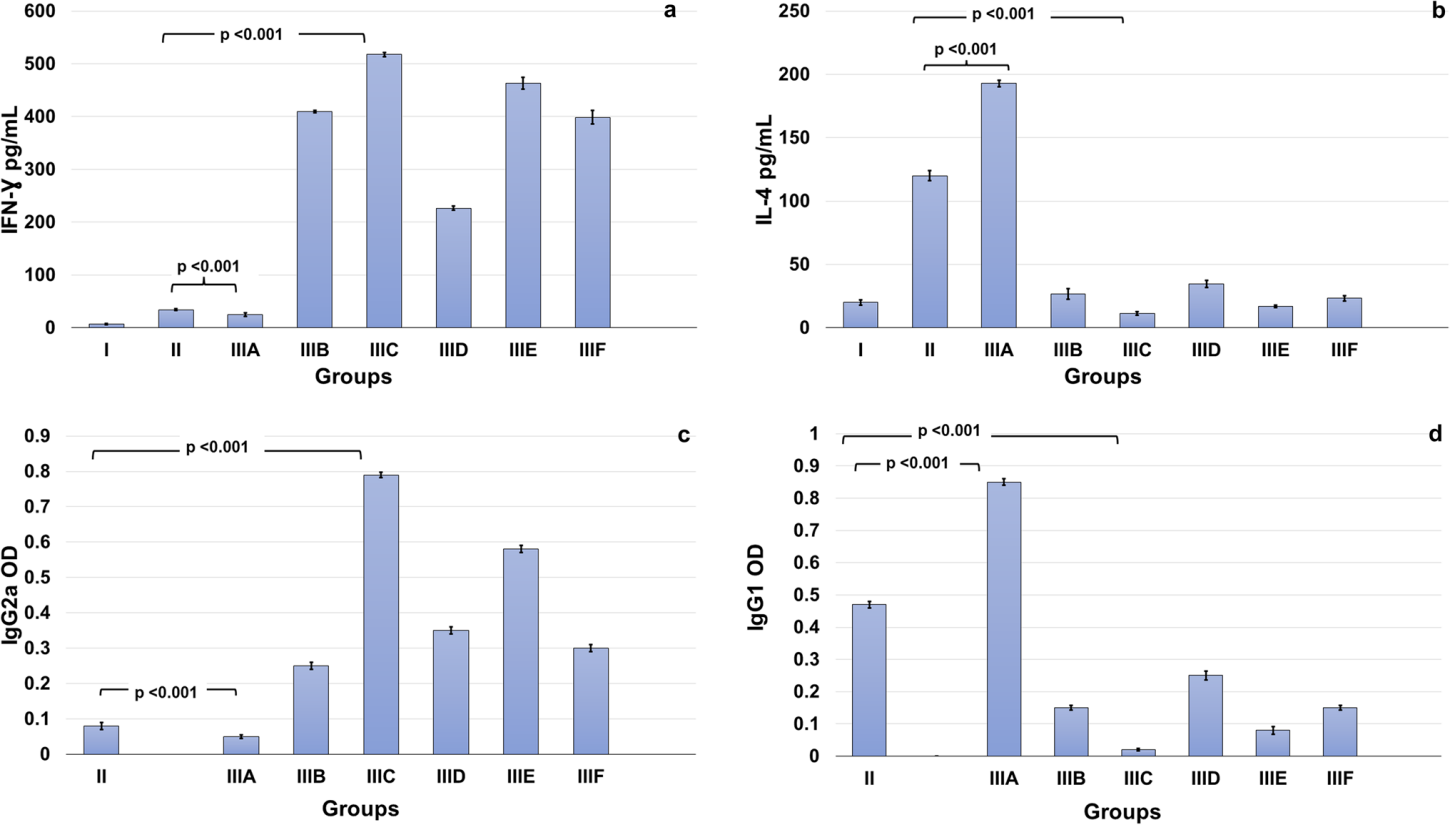
Column chart presentations of the biomarkers of Th1 and Th2 immune responses. **a** Levels of IFN-γ in popliteal LNs. The highest levels were detected in subgroup C followed by subgroup E, with statistically significant differences compared with other subgroups. **b** Levels of IL-4 in popliteal LNs. The lowest levels were detected in subgroup C followed by subgroup E, with statistically significant differences compared with other subgroups. **c** Serum IgG2a OD values. The highest levels were detected in subgroup C followed by subgroup E, with statistically significant differences compared with other subgroups. **d** Serum IgG1 OD values. The lowest levels were detected in subgroup C followed by subgroup E, with statistically significant differences compared with other groups

In contrast to the parasite burden, the highest level of IFN-γ was recorded in subgroup C (517.60 ± 3.64 pg/mL) followed by subgroup E (463.00 ± 11.09 pg/mL; *p* = 0.005), with statistically significant differences compared with the other subgroups (*p* < 0.001). The epitope of subgroup A demonstrated the lowest levels (24.60 ± 3.20 pg/mL) compared with the other subgroups, including the infected control (34.20 ± 1.92 pg/mL; *p* = 0.025), (Fig. 4a), whereas IL-4 demonstrated contrary results. The highest level of IL-4 was recorded in subgroup A (192.80 ± 2.58 pg/mL), with a statistically significant difference compared with the infected control (120.00 ± 3.80 pg/mL) and other subgroups (*p* < 0.001). The lowest level was recorded in subgroup C (11.20 ± 1.30 pg/mL) (Fig. 4b).

### Assessment of IgG2a and IgG1 response

Similar to IFN-γ results, the highest level of Th1-stimulated IgG2a release was detected in subgroup C (0.79 ± 0.008), which showed statistically significant differences compared with the other groups (*p* < 0.001). The second highest level of IgG2a was found in subgroup E (0.58 ± 0.01). The epitope of subgroup A showed the lowest IgG2a scores (0.05 ± 0.005) (Fig. 4c). Conversely, the Th2-stimulated IgG1 release was the highest in subgroup A (0.85 ± 0.01) (*p* < 0.001 for all groups) and lowest in subgroup C (0.02 ± 0.004) (Fig. 4d).

## Discussion

In this study, we aimed to evaluate peptide epitopes as vaccine candidates for CL—the most common form of leishmaniasis—for which there is no licensed vaccine. Because the chemotherapeutics used are toxic and infected patients in nonendemic areas are usually undiagnosed until irreversible scar formation, vaccination is the logical step to control this disfiguring disease.

In the past, leishmanization was widely used for many years in the Middle East as an effective way of vaccination. The high efficacy of this method is due to its similarity with natural infection that usually ends with a lifelong immunity to reinfection. It is practiced through intradermal inoculation of the virulent strain of *L. major* in hidden parts of the skin. The lifelong immunity that follows healing prevents the appearance of disfiguring scars on the face if reinfection occurs. However, it is no longer practiced due to safety and ethical issues, e.g., exacerbation and delayed healing of lesions at the site of inoculation occurred in some leishmanized individuals (Huang et al. 2015; Pacheco-Fernandez et al. 2021).

In addition to avoiding the hazards of live vaccines, the various advantages of peptide epitopes versus other vaccine candidates (Herrera-Najera et al. 2009) prompted us to perform this study. We selected peptides from *L. major* calpain because they are important for the survival and virulence of *Leishmania*. Such proteins stimulate potent immune responses and are a good choice as a vaccine candidate. Studies in which drugs targeted calpains achieved good therapeutic results (Ennes-Vidal et al. 2017; De Souza Araújo et al. 2018). Moreover, satisfactory results were reported when these were used as vaccine candidates in other organisms, e.g., *Schistosoma* (Rojo et al. 2017; Siddiqui and Siddiqui 2017). Because the immunogenicity of epitopes is generally lower than total protein antigens, its adsorption to an adjuvant is important to increase its antigenicity. We used Imject Alum as an adjuvant to benefit from its depot criterion. It binds strongly with the antigen, retaining it at the injection site for slower and continuous release (Raman et al. 2012; Ghimire 2015).

In silico prediction of calpain epitopes showed that, of the 203 predicted T-cell epitopes, strong affinities were exhibited by three highly antigenic, immunogenic, and highly conserved CD8 + T-cell epitopes (ARELAWATL, KHAGAIAHL, and LLRRLIVTK) and three CD4 + T-cell epitopes (NERARELAWATLAAD, DPAKHAGAIAHLESE, and TQHPVLLRRLIVTKE). Ochoa et al. (2019) reported that MHC-II molecules respond against extracellular proteins, in particular, small peptides to which they bind to activate the immune system.

Immunization with the TQHPVLLRRLIVTKE and KHAGAIAHL calpain epitopes induced TCM cell formation, which resulted in a potent stimulation of the protective Th1 immune response after *L. major* challenge. In a previous study, in silico predicted SARS-CoV-2 peptides were used individually or in combination to immunize female Balb/c mice. The immunized mice raised reactive antibodies against two out of six SARS-CoV-2 short peptides (Shehata et al. 2021).

The epitopes we designed were not restricted to CD4 + lymphocyte-directed epitopes. We developed CD8 + lymphocyte-directed epitopes because they are also crucial in preventing the spread of *L. major* infection and healing of lesions (Belkaid et al. 2002). Bioinformatics analyses of immunogenic T-cell epitopes of LeIF and PpSP15 proteins from *L. major* and sand fly saliva were used as model antigens to design a multiepitope vaccine for leishmaniasis. A complete set of 9-mer MHC-I and 15-mer MHC-II peptides was identified with a high affinity for antigenic epitopes that induce specific responses of CD8 + and CD4 + T-cells in BALB/c mice and humans (Bordbar et al. 2020). Salehi-Sangani et al. (2019) designed a multivalent DNA vaccine encoding the most immunogenic regions of *L. major* antigens, including thiol-specific antioxidant protein, *L. major* stress-inducible protein 1, *Leishmania* homologue of receptors for activated C kinase, and kinetoplastid membrane protein-11 in BALB/c mice. Although the DNA vaccine obtained from the immunogenic chimeric protein of *L. major* antigens could induce a high level of IFN-γ production, it protected mice against *L. major* partially.

We chose BALB/c mice as our animal model because of their natural genetic susceptibility to *L. major* infection. This strain lacks the ability to produce IL-12, which directs the immune response toward the protective Th1 lymphocytes; however, it develops the IL4-induced Th2 lymphocytes faster than resistant strains, causing progressive skin lesions and sometimes visceral invasion (Güler et al. 1996; von Stebut and Udey 2004). Thus, any degree of protection detected against the test epitopes was not the animal’s natural resistance to infection.

Healing in leishmaniasis depends on the generation of CD4 + Th1 cells, which produce IFN-γ, with subsequent activation of macrophages to kill intracellular parasites. This pathway ends with the formation of short-lived effector T-cells and long-lived central memory T-cells and skin-resident memory T-cells which are responsible for immunity to secondary infections. Therefore, a successful vaccine must induce long-lived memory T-cell formation that can be maintained without the continued presence of parasites (Glennie and Scott 2016).

We focused on TCM cells because among different memory cell types, TCM cells are responsible for mediating long-term immunity against *L. major* even in the absence of parasites (Zaph et al. 2004). The CD4 + TCM cell type was chosen because it is crucial for the generation of functional and protective antigen-specific CD8 + T-cells and B-cells in addition to the protective Th1 cells (MacLeod et al. 2010). This could explain the marked increase in Th1 biomarkers (i.e., IFN-γ and IgG2a) in subgroups C and E, which had the highest percentages of TCM cells. The role of epitope-induced TCM cells was confirmed in subgroup A. This group scored the least TCM percentage and the lowest levels of IFN-γ and IgG2a. Contrarily, the levels of Th2 biomarkers (IL-4 and IgG1) in subgroup A were the highest among all groups. Thus, in absence of TCM cells, the epitope of subgroup A could not change the immunologic nature of BALB/c mice.

The predominant Th1 response, which was highest in subgroup C, can explain the marked reduction of parasite density in this subgroup. The high serum levels of IFN-γ secreted by Th1 lymphocytes, macrophages, and other immune cells are a well-known factor for resistance to *Leishmania* infection. We selected IFN-γ as the primary marker of activated Th1 response because it promotes IL-12 secretion and Th1 activation. Th1 cells reciprocally secrete IFN-γ in more significant amounts promoting macrophage activation at the lesion site, which in turn kills the intracellular *Leishmania* in a nitric oxide-dependent manner (Vanloubbeeck and Jones 2004; Scott and Novais 2016). Moreover, IFN-γ induces differentiation of CD8 + lymphocytes, which in turn secrete more IFN-γ. Due to similar IFN-dependent killing mechanisms (Uzonna et al. 2004), CD8 + lymphocytes should have contributed to parasite density reduction, especially in the subgroups immunized with the CD8 + targeting epitopes, e.g., subgroup E, which ranked second to subgroup C.

The humoral immune response of subgroup C was also of the protective type, IgG2a (Doroud et al. 2011). Shifting of the IgG2a/IgG1 ratio toward the IgG2a isotype is associated with a protective immune response because reduced IgG1 levels means lower IL-10 production from macrophages, and subsequently, reduced pathology and tissue damage (Miles et al. 2005; Rostamian et al. 2017). This can explain the reduced lesion size in this subgroup. The combination of TCM cells, protective Th1 lymphocytes, and IgG2a stimulation induced by the epitope of subgroup C meets the criteria for a good vaccine candidate (Pérez-Jiménez et al. 2006).

Because opposites make things manifest, the scores detected in the subgroup A, which showed the lowest percentages of TCM cells, showed importance of TCM cells. The marked predominance of the Th2 response, which increases pathology, was associated with the highest parasitic burden and skin lesion number in subgroup A compared with subgroup C. The elevation in IL-4 levels is a hallmark of the Th2 response. It drives Th0 differentiation toward the Th2 type and suppresses the development of Th1 lymphocytes. Elevated levels of IL-4 correlate positively with nonhealing and severity of *L. major* infection (Kropf et al. 2003; Poudel et al. 2020). Moreover, the increase in the IgG1 isotype may have contributed to this subgroup’s high parasite burden and impaired healing (Miles et al. 2005; Rostamian et al. 2017).

To our knowledge, this is the first published study to investigate calpain peptides as vaccine candidates for *L. major.* However, a calpain-based vaccine, against Sm-p80 protein, was used against *Schistosoma mansoni* infection and could stimulate both cellular and humoral immune responses with Th1 lymphocyte predominance (Rojo et al. 2017; Siddiqui and Siddiqui 2017). These findings are consistent with ours.

Based on our findings, the CD8 + lymphocyte-directed peptides demonstrated varying degrees of protection against *L. major* infection, which can be attributed to their ability to induce the formation of protective CD8 + lymphocytes that secret IFN-γ. These cells subsequently killed *L. major*, reduced parasite burden, and stimulated Th1 lymphocytes to kill more *Leishmania* and limit the tissue-damaging immune response, which is a vicious circle similar to that reported by Uzonna et al. (2004). Protection against *L. major* infection can also be attributed to the presence of TCM cells, which markedly increased in number in subgroup E and are crucial for the proliferation of CD8 + lymphocytes (MacLeod et al. 2010).

The absence of complete clearance of parasites with our epitopes is not a defect. The low levels of parasites are not pathological but stimulate effector and memory cells, which prevent incidence of harmful sequalae on exposure to second infection. This is a goal of an effective vaccine candidate (Glennie and Scott 2016).

## Conclusion

Our findings reveal that the calpain peptide epitopes TQHPVLLRRLIVTKE and KHAGAIAHL directed toward CD4 + and CD8 + lymphocytes, respectively, provided the best protection against *L. major* infection. They upregulated TCM lymphocytes, and subsequently, the protective Th1 response and its biomarkers (i.e., IFN-γ and IgG2a) while downregulating the pathogenic Th2 lymphocytes. This is a pilot study that identifies calpain peptides as potential vaccine candidates because they meet important criteria, control infection, and generate memory cells.

### Limitations of the study

Our study has some limitations. First, the absence of a licensed vaccine prevents us from comparing our results with the designed epitopes with that of the vaccine. Second, more cytokines and immune markers, e.g., IL-10, must be assessed to give a precise view of the resultant immune response.

## Data Availability

All data generated or analyzed during this study are included in this published article (and its supplementary information files).
